# Supplementary material for: Deletion of wfs1 Impairs Oligodendrocyte Precursor Cells Dorsal Distribution and Myelination Through the wfs1-hmgcs1 Axis in Zebrafish
Source: Biology (Basel). 2026 Aug 16;15(16):1402. doi: 10.3390/biology15161402 (PMC13510151; doi:10.3390/biology15161402)
Supplement: Supplementary file 1 [file biology-15-01402-s001.zip › File S1-Transcriptome sequencing data and differentially expressed genes (DEGs) in wfs1-- zebrafish at 5 dpf/2.KEGG/wfs1KOvsWT/GSEA/WT/RIBOSOME/RIBOSOME(DRE03010).html]

Details for gene set RIBOSOME(DRE03010)[GSEA]

|  || Dataset | deg.sample |
| Phenotype | sample.cls |
| Upregulated in class | WT |
| GeneSet | RIBOSOME(DRE03010) |
| Enrichment Score (ES) | -0.21864988 |
| Normalized Enrichment Score (NES) | -0.8266607 |
| Nominal p-value | 0.54583335 |
| FDR q-value | 0.8195717 |
| FWER p-Value | 1.0 |
Table: GSEA Results Summary

  

Fig 1: Enrichment plot: RIBOSOME(DRE03010)      
 Profile of the Running ES Score & Positions of GeneSet Members on the Rank Ordered List

  

| PROBE | DESCRIPTION (from dataset) | GENE SYMBOL | GENE\_TITLE | RANK IN GENE LIST | RANK METRIC SCORE | RUNNING ES | CORE ENRICHMENT || 1 | 336712\_2 | rpl10 |  |  | 15 | 4.958 | 0.0641 | No |
| 2 | 415196\_1 | rpl5b |  |  | 56 | 4.865 | 0.1262 | No |
| 3 | 327089 | rpl12 |  |  | 637 | 2.707 | 0.1432 | No |
| 4 | 560828\_1 | rpl13a |  |  | 1027 | 2.069 | 0.1579 | No |
| 5 | 553284\_1 | mrpl1 |  |  | 1174 | 1.925 | 0.1783 | No |
| 6 | 337007\_1 | rps12 |  |  | 1987 | 1.372 | 0.1706 | No |
| 7 | 556556\_1 | rpl32 |  |  | 3578 | 0.914 | 0.1324 | No |
| 8 | 550564\_2 | faub |  |  | 4887 | 0.696 | 0.1002 | No |
| 9 | 337148\_1 | rps15 |  |  | 4895 | 0.695 | 0.1091 | No |
| 10 | 337240\_1 | rps3a |  |  | 5130 | 0.664 | 0.1103 | No |
| 11 | 553799\_1 | mrpl30 |  |  | 5152 | 0.661 | 0.1183 | No |
| 12 | 560828 | rpl13a |  |  | 5601 | 0.626 | 0.1123 | No |
| 13 | 323305\_1 | rpl10a |  |  | 5692 | 0.619 | 0.1175 | No |
| 14 | 100000836 | rps8b |  |  | 6177 | 0.570 | 0.1097 | No |
| 15 | 195819\_1 | rpl36a |  |  | 6943 | 0.496 | 0.0921 | No |
| 16 | 336713\_1 | rpl30 |  |  | 7136 | 0.479 | 0.0923 | No |
| 17 | 554098 | mrpl28 |  |  | 8680 | 0.389 | 0.0487 | No |
| 18 | 550391 | mrpl12 |  |  | 8811 | 0.380 | 0.0495 | No |
| 19 | 336713 | rpl30 |  |  | 8901 | 0.374 | 0.0516 | No |
| 20 | 337810 | rps27a |  |  | 9031 | 0.365 | 0.0523 | No |
| 21 | 337148 | rps15 |  |  | 9398 | 0.341 | 0.0452 | No |
| 22 | 101886382\_1 | si:dkey-159f12.2 |  |  | 9781 | 0.317 | 0.0373 | No |
| 23 | 336702 | rpl9 |  |  | 10126 | 0.294 | 0.0303 | No |
| 24 | 677743 | zgc:136826 |  |  | 10676 | 0.262 | 0.0164 | No |
| 25 | 337810\_1 | rps27a |  |  | 10695 | 0.261 | 0.0192 | No |
| 26 | 415142 | mrpl23 |  |  | 10992 | 0.244 | 0.0131 | No |
| 27 | 735250 | rpl22l1 |  |  | 11268 | 0.229 | 0.0074 | No |
| 28 | 337240 | rps3a |  |  | 11317 | 0.226 | 0.0088 | No |
| 29 | 436759 | rpl38 |  |  | 11697 | 0.206 | -0.0004 | No |
| 30 | 619204 | mrps7 |  |  | 11914 | 0.195 | -0.0047 | No |
| 31 | 394121 | rps10 |  |  | 11924 | 0.195 | -0.0024 | No |
| 32 | 100142645 | mrpl22 |  |  | 12586 | 0.160 | -0.0212 | No |
| 33 | 449547 | rps4x |  |  | 12876 | 0.147 | -0.0284 | No |
| 34 | 336568\_1 | rpl21 |  |  | 13402 | 0.121 | -0.0434 | No |
| 35 | 415168 | rpl39 |  |  | 13403 | 0.121 | -0.0418 | No |
| 36 | 768305 | mrpl21 |  |  | 13541 | 0.114 | -0.0446 | No |
| 37 | 378961 | rpl13 |  |  | 13968 | 0.094 | -0.0568 | No |
| 38 | 556272 | mrps11 |  |  | 14684 | 0.061 | -0.0786 | No |
| 39 | 415159 | rpl37 |  |  | 14774 | 0.057 | -0.0806 | No |
| 40 | 336283 | mrpl3 |  |  | 15030 | 0.045 | -0.0881 | No |
| 41 | 793878 | si:dkey-17e16.10 |  |  | 15050 | 0.043 | -0.0881 | No |
| 42 | 336719 | rplp1 |  |  | 15083 | 0.042 | -0.0886 | No |
| 43 | 751640 | mrpl20 |  |  | 15088 | 0.042 | -0.0882 | No |
| 44 | 565937 | mrps10 |  |  | 15627 | 0.016 | -0.1049 | No |
| 45 | 101886382 | si:dkey-159f12.2 |  |  | 15798 | 0.007 | -0.1102 | No |
| 46 | 393738 | rps27.2 |  |  | 15860 | 0.004 | -0.1121 | No |
| 47 | 562703\_1 | mrps5 |  |  | 15867 | 0.004 | -0.1122 | No |
| 48 | 751681 | mrps9 |  |  | 16161 | -0.007 | -0.1213 | No |
| 49 | 100073334 | mrps21 |  |  | 16303 | -0.011 | -0.1256 | No |
| 50 | 569841 | mrps18c |  |  | 16368 | -0.013 | -0.1275 | No |
| 51 | 336712\_1 | rpl10 |  |  | 16499 | -0.017 | -0.1314 | No |
| 52 | 445040 | rpl29 |  |  | 16793 | -0.026 | -0.1403 | No |
| 53 | 335539 | rpl23a |  |  | 16800 | -0.026 | -0.1401 | No |
| 54 | 58101 | rplp0 |  |  | 16870 | -0.029 | -0.1419 | No |
| 55 | 406387 | rpl26 |  |  | 16988 | -0.033 | -0.1452 | No |
| 56 | 751730 | mrpl32 |  |  | 17071 | -0.035 | -0.1473 | No |
| 57 | 493604 | mrpl16 |  |  | 17099 | -0.036 | -0.1477 | No |
| 58 | 336764 | rps15a |  |  | 17144 | -0.038 | -0.1486 | No |
| 59 | 394166 | rps21 |  |  | 17301 | -0.045 | -0.1529 | No |
| 60 | 445053 | rpl15 |  |  | 17540 | -0.055 | -0.1597 | No |
| 61 | 394194 | rps24 |  |  | 17659 | -0.061 | -0.1626 | No |
| 62 | 322571 | rpl3 |  |  | 17670 | -0.061 | -0.1621 | No |
| 63 | 192297 | rps5 |  |  | 17673 | -0.061 | -0.1614 | No |
| 64 | 323305 | rpl10a |  |  | 17706 | -0.063 | -0.1616 | No |
| 65 | 405888 | rpl36 |  |  | 17803 | -0.068 | -0.1637 | No |
| 66 | 553771 | mrpl4 |  |  | 17896 | -0.071 | -0.1657 | No |
| 67 | 571648 | mrpl2 |  |  | 17929 | -0.073 | -0.1657 | No |
| 68 | 325618 | rpl27 |  |  | 17962 | -0.074 | -0.1658 | No |
| 69 | 572109 | zgc:171772 |  |  | 18112 | -0.082 | -0.1694 | No |
| 70 | 407690 | rps8a |  |  | 18430 | -0.095 | -0.1782 | No |
| 71 | 394036 | rpl28 |  |  | 18436 | -0.095 | -0.1771 | No |
| 72 | 552972 | mrpl34 |  |  | 18517 | -0.100 | -0.1783 | No |
| 73 | 337090 | rpl4 |  |  | 18607 | -0.104 | -0.1798 | No |
| 74 | 445028 | rps6 |  |  | 18723 | -0.109 | -0.1820 | No |
| 75 | 394027 | rpsa |  |  | 18829 | -0.115 | -0.1838 | No |
| 76 | 393715 | rps26l |  |  | 18900 | -0.118 | -0.1844 | No |
| 77 | 335629 | rplp2 |  |  | 18938 | -0.119 | -0.1841 | No |
| 78 | 447945 | mrpl17 |  |  | 19123 | -0.128 | -0.1882 | No |
| 79 | 327089\_1 | rpl12 |  |  | 19279 | -0.134 | -0.1913 | No |
| 80 | 436760 | rpl35a |  |  | 19470 | -0.143 | -0.1955 | No |
| 81 | 767765 | rps16 |  |  | 19495 | -0.143 | -0.1944 | No |
| 82 | 791211 | mrps2 |  |  | 19546 | -0.145 | -0.1940 | No |
| 83 | 415229 | rpl11 |  |  | 19687 | -0.152 | -0.1965 | No |
| 84 | 406686 | rps11 |  |  | 19828 | -0.158 | -0.1988 | No |
| 85 | 394035 | rpl18a |  |  | 19874 | -0.160 | -0.1981 | No |
| 86 | 445150 | mrpl19 |  |  | 19942 | -0.164 | -0.1981 | No |
| 87 | 336710 | rpl7 |  |  | 20042 | -0.168 | -0.1991 | No |
| 88 | 406565 | rps2 |  |  | 20146 | -0.173 | -0.2001 | No |
| 89 | 641289 | uba52 |  |  | 20156 | -0.173 | -0.1981 | No |
| 90 | 553709 | mrpl27 |  |  | 20420 | -0.184 | -0.2040 | No |
| 91 | 393818 | rps17 |  |  | 20814 | -0.205 | -0.2137 | No |
| 92 | 405889 | rps29 |  |  | 20847 | -0.207 | -0.2120 | No |
| 93 | 336550 | rps3 |  |  | 20858 | -0.207 | -0.2096 | No |
| 94 | 553555 | mrpl13 |  |  | 20864 | -0.208 | -0.2071 | No |
| 95 | 554155 | rps27.1 |  |  | 21092 | -0.220 | -0.2114 | No |
| 96 | 192300 | rps18 |  |  | 21324 | -0.232 | -0.2156 | Yes |
| 97 | 100000341 | rps23 |  |  | 21355 | -0.234 | -0.2135 | Yes |
| 98 | 436829 | mrpl11 |  |  | 21496 | -0.241 | -0.2148 | Yes |
| 99 | 326961 | rpl5a |  |  | 21521 | -0.242 | -0.2124 | Yes |
| 100 | 541327 | zgc:114188 |  |  | 21669 | -0.250 | -0.2138 | Yes |
| 101 | 337240\_2 | rps3a |  |  | 21701 | -0.251 | -0.2115 | Yes |
| 102 | 393710 | faua |  |  | 21705 | -0.252 | -0.2083 | Yes |
| 103 | 336641 | rpl17 |  |  | 21749 | -0.254 | -0.2063 | Yes |
| 104 | 103910031 | LOC103910031 |  |  | 21849 | -0.259 | -0.2061 | Yes |
| 105 | 100093703 | mrpl36 |  |  | 21869 | -0.260 | -0.2033 | Yes |
| 106 | 114420 | rps9 |  |  | 21901 | -0.262 | -0.2009 | Yes |
| 107 | 337010 | rpl7a |  |  | 22076 | -0.271 | -0.2028 | Yes |
| 108 | 336812 | rpl23 |  |  | 22112 | -0.274 | -0.2004 | Yes |
| 109 | 553580 | mrps17 |  |  | 22155 | -0.276 | -0.1981 | Yes |
| 110 | 751639 | mrps14 |  |  | 22158 | -0.276 | -0.1946 | Yes |
| 111 | 677758 | rpl31 |  |  | 22426 | -0.291 | -0.1992 | Yes |
| 112 | 558080 | mrps18a |  |  | 22433 | -0.292 | -0.1956 | Yes |
| 113 | 406489 | rpl19 |  |  | 22504 | -0.296 | -0.1939 | Yes |
| 114 | 336702\_1 | rpl9 |  |  | 22596 | -0.301 | -0.1929 | Yes |
| 115 | 406485 | rps20 |  |  | 22597 | -0.301 | -0.1889 | Yes |
| 116 | 557397 | rpl22 |  |  | 22625 | -0.303 | -0.1858 | Yes |
| 117 | 336727 | rpl6 |  |  | 22814 | -0.314 | -0.1877 | Yes |
| 118 | 393725 | rps7 |  |  | 22991 | -0.327 | -0.1890 | Yes |
| 119 | 393788 | rps25 |  |  | 23188 | -0.340 | -0.1907 | Yes |
| 120 | 558312 | rplp2l |  |  | 23342 | -0.350 | -0.1910 | Yes |
| 121 | 445038 | rpl18 |  |  | 23353 | -0.351 | -0.1867 | Yes |
| 122 | 393686 | rpl8 |  |  | 23383 | -0.353 | -0.1830 | Yes |
| 123 | 767758 | mrpl35 |  |  | 23395 | -0.354 | -0.1788 | Yes |
| 124 | 394097 | rpl34 |  |  | 23447 | -0.357 | -0.1757 | Yes |
| 125 | 406307 | rps28 |  |  | 23515 | -0.362 | -0.1731 | Yes |
| 126 | 436674 | mrpl24 |  |  | 23652 | -0.371 | -0.1726 | Yes |
| 127 | 114420\_1 | rps9 |  |  | 23809 | -0.381 | -0.1725 | Yes |
| 128 | 393723 | rps19 |  |  | 23966 | -0.390 | -0.1724 | Yes |
| 129 | 323365 | rpl14 |  |  | 24141 | -0.402 | -0.1726 | Yes |
| 130 | 336662 | rps26 |  |  | 24198 | -0.406 | -0.1691 | Yes |
| 131 | 192299 | rpl35 |  |  | 24642 | -0.425 | -0.1775 | Yes |
| 132 | 394122 | mrpl14 |  |  | 24677 | -0.428 | -0.1730 | Yes |
| 133 | 336568 | rpl21 |  |  | 24981 | -0.444 | -0.1768 | Yes |
| 134 | 336726 | rpl27a |  |  | 25290 | -0.466 | -0.1804 | Yes |
| 135 | 751746 | mrps6 |  |  | 25380 | -0.473 | -0.1771 | Yes |
| 136 | 336687 | rps14 |  |  | 25577 | -0.492 | -0.1768 | Yes |
| 137 | 415168\_1 | rpl39 |  |  | 25653 | -0.498 | -0.1727 | Yes |
| 138 | 436758 | mrps15 |  |  | 25763 | -0.508 | -0.1695 | Yes |
| 139 | 445041 | mrpl15 |  |  | 25767 | -0.509 | -0.1630 | Yes |
| 140 | 415169 | rps13 |  |  | 26134 | -0.540 | -0.1675 | Yes |
| 141 | 195819 | rpl36a |  |  | 26281 | -0.554 | -0.1649 | Yes |
| 142 | 768187 | mrpl18 |  |  | 26603 | -0.588 | -0.1673 | Yes |
| 143 | 406266 | rsl24d1 |  |  | 26740 | -0.603 | -0.1638 | Yes |
| 144 | 415142\_1 | mrpl23 |  |  | 26778 | -0.607 | -0.1570 | Yes |
| 145 | 192301 | rpl24 |  |  | 27310 | -0.650 | -0.1653 | Yes |
| 146 | 572170 | mrpl9 |  |  | 27631 | -0.691 | -0.1664 | Yes |
| 147 | 415196 | rpl5b |  |  | 27791 | -0.714 | -0.1621 | Yes |
| 148 | 541330\_1 | mrpl10 |  |  | 28190 | -0.770 | -0.1646 | Yes |
| 149 | 556556\_2 | rpl32 |  |  | 28898 | -0.898 | -0.1752 | Yes |
| 150 | 562703 | mrps5 |  |  | 28958 | -0.910 | -0.1652 | Yes |
| 151 | 553799 | mrpl30 |  |  | 29369 | -1.020 | -0.1648 | Yes |
| 152 | 327089\_2 | rpl12 |  |  | 29463 | -1.050 | -0.1541 | Yes |
| 153 | 556556 | rpl32 |  |  | 29555 | -1.079 | -0.1429 | Yes |
| 154 | 337007 | rps12 |  |  | 29559 | -1.079 | -0.1289 | Yes |
| 155 | 100536515 | mrpl33 |  |  | 29942 | -1.222 | -0.1250 | Yes |
| 156 | 336712 | rpl10 |  |  | 31041 | -2.016 | -0.1334 | Yes |
| 157 | 450009\_1 | mrps16 |  |  | 31141 | -2.138 | -0.1087 | Yes |
| 158 | 553284 | mrpl1 |  |  | 31242 | -2.310 | -0.0817 | Yes |
| 159 | 550564\_1 | faub |  |  | 31359 | -2.548 | -0.0522 | Yes |
| 160 | 768187\_1 | mrpl18 |  |  | 31397 | -2.643 | -0.0189 | Yes |
| 161 | 554098\_2 | mrpl28 |  |  | 31398 | -2.645 | 0.0155 | Yes |
Table: GSEA details [plain text format]

  

Fig 2: RIBOSOME(DRE03010)      
 Blue-Pink O' Gram in the Space of the Analyzed GeneSet

  

Fig 3: RIBOSOME(DRE03010): Random ES distribution      
 Gene set null distribution of ES for **RIBOSOME(DRE03010)**
